# Impact of gender on the prevalence and extent of microvascular obstruction after st-elevation myocardial infarction

**DOI:** 10.1186/1532-429X-13-S1-P103

**Published:** 2011-02-02

**Authors:** Suzanne de Waha, Ingo Eitel, Steffen Desch, Georg Fuernau, Philipp Lurz, Matthias Grothoff, Matthias Gutberlet, Gerhard Schuler, Holger Thiele

**Affiliations:** 1 University of Leipzig – Heart Center, Leipzig, Germany

## Background

Baseline characteristics of women suffering from ST-elevation myocardial infarction (STEMI) diverge compared to men. Therefore the presence and extent of microvascular obstruction (MO), which has been shown to be a prognostic marker for adverse clinical outcome after STEMI, might differ between genders. Up to date gender differences of MO after STEMI have not been evaluated.

## Methods

STEMI patients reperfused by primary angioplasty (n=423) within 12 hours after symptom onset underwent contrast-enhanced-MRI at a median of 3 (interquartile range [IQR] 2;4) days after the index event. MO was measured 15 minutes after gadolinium injection with late enhancement sequences and evaluated qualitatively and quantitatively (as percentage of left ventricular mass [%LV]).

## Results

A total of 105 women and 318 men were analysed (24.6 vs. 76.4%, p<0.001). In comparison to men, women were significantly older (71.5 [IQR 63.4;77.0] vs. 63.7 [IQR 54.0;71.0] years, p<0.001), displayed longer symptom-onset-to-reperfusion times (241 [IQR 156;389] vs. 196 [IQR 132;337] minutes, p=0.04), a higher prevalence of diabetes mellitus (35.9 vs. 23.3%, p=0.02) and arterial hypertension (76.7 vs. 65.6%, p=0.04). Complete epicardial reperfusion defined as post-PCI TIMI-flow III (95.2 vs. 95.2%, p=1.0) did not differ significantly between genders.

The prevalence and extent of MO, infarct size and left ventricular ejection fraction were similar in women and men (MO presence: 65.4 vs. 71.9%, p=0.22 / MO extent: 0.59 [IQR 0;1.24] vs. 0.71 [IQR 0;2.0] %LV, p=0.47; infarct size: 13.9 [IQR 5.8;25.6] vs. 18.4 [IQR 8.7;29.0] %LV, p=0.14; ejection fraction: 52.2 [IQR 42.6;60.0] vs. 49.4 [40.7;57.9] %, p=0.16).

## Conclusion

Despite longer ischemic time and a more disadvantageous cardiovascular risk profile in women the prevalence and extent of MO do not differ between genders.

